# Dr. W. C. Wardlaw

**Published:** 1893-11

**Authors:** 


### Obituary.

### DR. W. C. WARDLAW.

Dr. W. C. Wardlaw died at his home, Augusta, Ga., September 3, 1893.

Very few members of the Southern wing of the dental profession were as well known as the subject of this sketch. He was constantly active, when in health, for the advancement of his chosen calling, and no labor seemed too great, or no distance too far for him to travel to meet his professional friends in the two annual conventions in which he was always an active participant,—the Southern and American Dental Associations.

His last effort, just previous to his death, was to attend the Dental Congress at Chicago, and it was the writer's painful, though gratifying, privilege to meet him there, and though he seemed feeble, yet he presented the old time interest.

Dr. Wardlaw's work has been always of a quiet character, but none the less effective for the advancement of his profession. He will be seriously missed North and South, and in both sections will be sincerely mourned.

He had been in poor health for a long time, and confined to the house for four months of that period. Subsequently to this he partially recovered his strength, and made the trip to Chicago under the hope that change of scene would be beneficial. He completed his dental studies at the Pennsylvania College of Dental Surgery, graduating from that institution in 1866.

He was elected in August to the position of Dean in the Atlanta Dental College.

The immediate cause of death was congestion of the brain.

Dr. Wardlaw was twice married, and leaves eight children to mourn a good father, faithful friend, and a consistent professional man.

